# The Effect of Virtual Laboratories on the Academic Achievement of Undergraduate Chemistry Students: Quasi-Experimental Study

**DOI:** 10.2196/64476

**Published:** 2024-11-15

**Authors:** Hiwot Bazie, Bekele Lemma, Anteneh Workneh, Ashebir Estifanos

**Affiliations:** 1 Department of Chemistry College of Natural and Computational Science Hawassa University Hawassa Ethiopia; 2 Department of Educational Leadership and Management College of Education Hawassa University Hawassa Ethiopia

**Keywords:** virtual laboratory, practical chemistry, student achievement, undergraduate student, Dilla University, simulation, chemistry education

## Abstract

**Background:**

Experimentation is crucial in chemistry education as it links practical experience with theoretical concepts. However, practical chemistry courses typically rely on real laboratory experiments and often face challenges such as limited resources, equipment shortages, and logistical constraints in university settings. To address these challenges, computer-based laboratories have been introduced as a potential solution, offering electronic simulations that replicate real laboratory experiences.

**Objective:**

This study examines the effect of virtual laboratories on the academic achievement of undergraduate chemistry students and evaluates their potential as a viable alternative or complement to traditional laboratory-based instruction.

**Methods:**

A quasi-experimental design was implemented to examine the cause-and-effect relationship between instructional methods and student outcomes. The study involved 60 fourth-year BSc chemistry students from Dilla University, divided into 3 groups: a real laboratory group (n=20), which performed real laboratory experiments; a virtual group (n=20), which used virtual laboratory simulations; and a lecture group (n=20), which received lecture-based instruction. Quantitative data were collected through tests administered before and after the intervention to assess academic performance. The data analysis used descriptive and inferential statistics, such as means and SDs, 1-way ANOVA, the Tukey honestly significant difference test, and independent-sample *t* tests (2-tailed), with a *P* value of .05 set for determining statistical significance.

**Results:**

Before the intervention, the results indicated no significant differences in academic achievement among the 3 groups (*P*=.99). However, after the intervention, notable differences were observed in student performance across the methods. The real laboratory group had the highest mean posttest score (mean 62.6, SD 10.7), followed by the virtual laboratory group (mean 55.5, SD 6.8) and the lecture-only group, which had the lowest mean score (mean 43.7, SD 11.5). ANOVA results confirmed significant differences between the groups (*F*_2,57_=18.429; *P*<.001). The Tukey post hoc test further revealed that the real laboratory group significantly outperformed the lecture-only group (mean difference 18.88; *P*<.001), while the virtual laboratory group also performed significantly better than the lecture-only group (mean difference 11.7; *P*=.001). However, no statistically significant difference was found between the real laboratory and virtual laboratory groups (mean difference 7.12; *P*=.07). In addition, gender did not significantly influence performance in the virtual laboratory group (*P*=.21), with no substantial difference in posttest scores between male and female students.

**Conclusions:**

These findings suggest that computer-based laboratories are a viable and effective alternative when real laboratories are unavailable, enhancing learning outcomes when compared with traditional lecture-based methods. Therefore, universities should consider integrating computer-based laboratories into their practical chemistry curricula to provide students with interactive and engaging learning experiences, especially when physical laboratories are inaccessible.

## Introduction

### Background

Experimentation plays a vital role in chemistry education, enhancing student learning and achievement by making content more meaningful. Since the 19th century, the importance of hands-on laboratory instruction has been recognized for helping students explore and understand the complexities of the natural world [1,2]. Through experimentation, students develop essential scientific skills, such as observation, inference, prediction, communication, analysis, and critical thinking, which are key to scientific inquiry [3-5]. These skills also promote analytical reasoning and active participation in scientific inquiry. Furthermore, experiments help translate abstract concepts into practical understanding, reinforce classroom learning, and connect chemistry to real-world applications [6-8]. Recognizing these benefits, Ethiopian universities included 1 or 2 credits of practical chemistry courses in their curricula. These courses enable students to deepen their knowledge through hands-on experiments by analyzing chemical compounds, exploring inorganic reactions, investigating organic molecules, and applying fundamental chemical principles. This practical approach connects theoretical knowledge with real-world applications, promotes independent exploration, and substantially improves students’ laboratory skills.

Although laboratory experiments benefit both teachers and students, many chemistry experiments are not conducted in low-income countries such as Ethiopia owing to limited resources, safety concerns, and logistical problems [5,9]. In Ethiopian universities, the high cost of chemicals and equipment is a barrier, resulting in students being able to perform only a small number of experiments. This scarcity of exposure to practical experience reduces their interest in chemistry, impedes their ability to teach effectively, and hinders employment in industrial positions that demand hands-on skills. Scholars suggest that computer-based (ie, virtual) laboratories could address these issues by offering a viable alternative to traditional laboratory experiments when resources are inadequate [10].

Computer-based laboratories are crucial for both instructors and learners, offering clear instructions and visual representations of natural phenomena [1,11]. According to Tatli and Ayas [9] and Asare et al [12] virtual laboratories introduce innovative strategies that support high-level skills, such as problem-solving and experimental design. They capture students’ attention; motivate them; and enhance collaborative discussions among students, peers, and teachers. Virtual laboratories provide an engaging learning experience by allowing students to interact with equipment, collect and analyze data, prepare experimental reports, and enhance their cognitive skills [4,13,14]. Al Mulla and Ali [15] and Lewis [16] found that virtual simulations improved students’ knowledge and confidence in laboratory work by providing flexible access.

Given the significance of computer-based laboratories in science education, particularly in chemistry, they have been implemented worldwide at all levels, from primary school to university. Numerous studies have explored the application of virtual laboratories in chemistry education across different regions. For instance, Latifah et al [17] found that students in a virtual chemistry laboratory (experimental group) achieved higher cognitive learning outcomes than those in the real laboratory group. However, their study focused on overall cognitive learning achievement without examining specific cognitive skills [10] and investigated the impact of virtual experimental platforms on students’ self-efficacy in chemistry laboratories. The overall result revealed a positive effect. However, the study did not explore how different student demographics might influence the effectiveness of these platforms. Akomaye [18] examined the effect of virtual laboratory simulations on senior secondary school students’ understanding of science process skills in practical chemistry and found significant enhancement in their skills. However, this study did not address the impact on university students’ science process skills. A study on virtual reality in the chemistry laboratory showed positive effects on students’ self-efficacy, interest, and self-concept, as well as a reduction in laboratory anxiety [19]. However, it did not explore the long-term impact on learning achievements or engagement in real laboratory settings.

While real laboratory experiments are undeniably crucial for developing cognitive, affective, and psychomotor learning domains, fully implementing them in low-income countries such as Ethiopia poses the aforementioned challenges. Hence, several educational sectors have started integrating technology-based education to overcome the challenges that are related to the teaching and learning process. Recognizing the need to enhance teaching and learning, the Ethiopian government has integrated information and communication technology into its educational policy [20]. The Ethiopian Education Development Road Map (2018 to 2030) emphasizes the importance of improving technology use in universities for both academic and research purposes. As a result, many Ethiopian universities have incorporated technology into their teaching and learning processes [21]. Despite the advances in information and communication technology, the extent to which virtual laboratories are implemented in Ethiopian universities remains unclear, particularly in contexts where real laboratory experiments face numerous challenges. Moreover, no research has been conducted so far in Ethiopian universities to assess the impact of virtual laboratories on students’ academic achievement. This study was thus designed to examine the effect of virtual chemistry laboratories on the practical chemistry achievements of undergraduate students at Dilla University. The study specifically examined the differences in academic achievement among students who learned practical chemistry through virtual laboratories, those who used real laboratories, and those who received only lecture-based instruction.

### Hypotheses

The study formulated the following hypotheses:

- Hypothesis 1: there is no significant difference in academic achievement between students who learned practical chemistry through a virtual laboratory compared with those who learned in real laboratories and those who attended only lectures at Dilla University.
- Hypothesis 2: there is no significant difference in practical chemistry achievement scores between male and female students exposed to the virtual chemistry laboratory at Dilla University.

### Objectives

The general objective of this study was to examine the effect of virtual laboratories on the practical chemistry achievements of undergraduate students at Dilla University. The specific objectives of this study were as follows: (1) to investigate the academic achievement of students who learned practical chemistry using virtual laboratories compared with those who attended real laboratories or lectures only at Dilla University and (2) to explore any significant differences in practical chemistry achievement scores between male and female students exposed to the virtual chemistry laboratory at Dilla University.

### Theoretical Framework

The theoretical framework for examining the impact of virtual laboratories on undergraduate students’ academic achievement in practical chemistry at Dilla University includes several key theories. The Technology Acceptance Model suggests that perceived usefulness and ease of use are critical factors in technology adoption [22]. In the context of virtual laboratories, the Technology Acceptance Model evaluates students’ perceptions to determine how these factors affect their learning outcomes in practical chemistry. The constructivist learning theory underscores the significance of active engagement, inquiry, problem-solving, and collaboration in the construction of knowledge [15,23]. Virtual laboratories align with this theory by providing hands-on experimentation and fostering active participation. The experiential learning theory highlights the importance of concrete experiences and reflective observation in understanding concepts [24]. Virtual laboratories facilitate experiential learning for chemistry students, offering interactive and reflective activities that promote deeper comprehension. Finally, the cognitive load theory recommends that instructional design should manage cognitive load to enhance learning [25]. Virtual laboratories are designed to efficiently handle cognitive load, thereby improving students’ understanding and retention of chemistry concepts.

### Conceptual Framework

**Figure 1:**
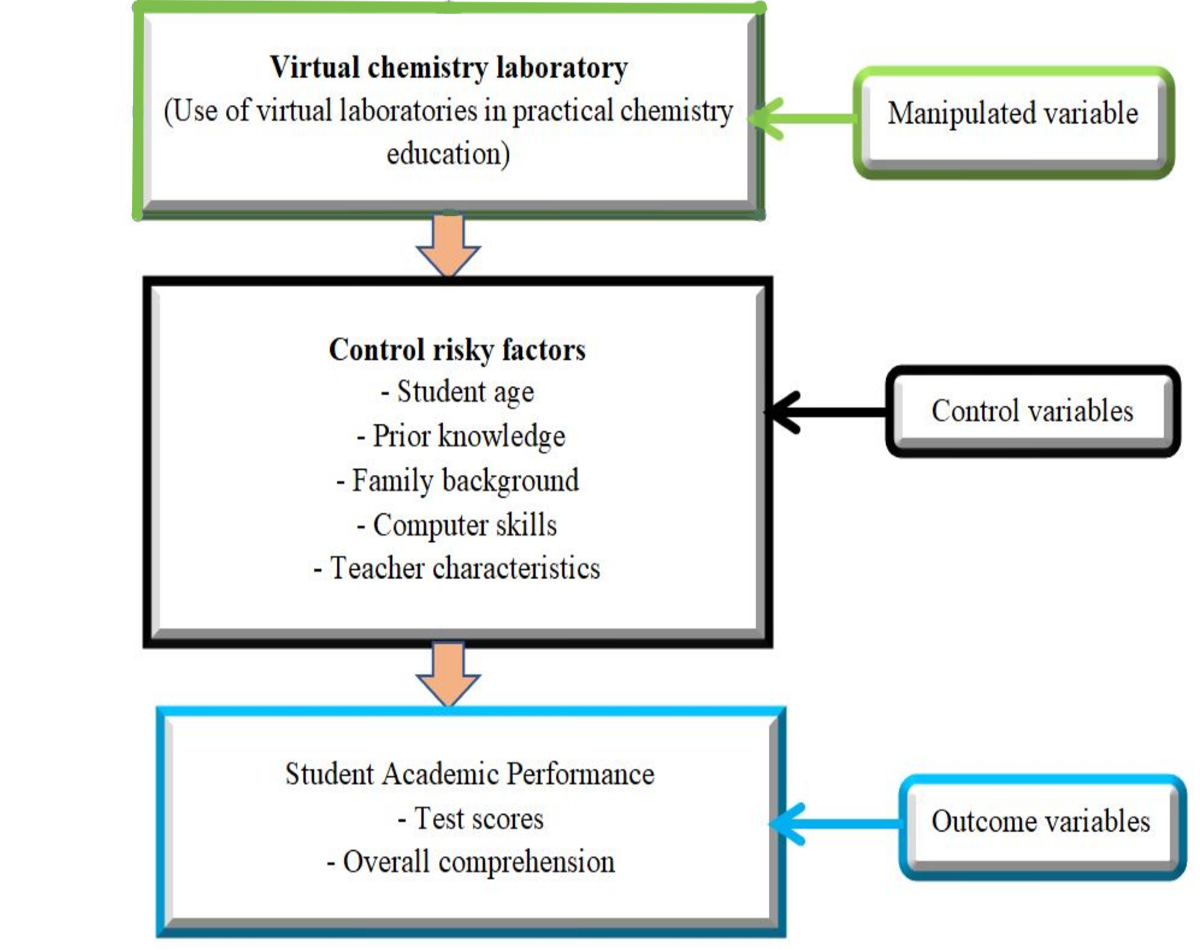
Conceptual framework of the study.

As depicted in Figure 1, the conceptual framework shows the relationship between the manipulated variable (virtual chemistry laboratory) and the outcome variable (students’ academic performance). The virtual chemistry laboratory enhances practical chemistry education by providing a flexible, safe, and interactive environment for conducting experiments and developing skills, potentially improving student achievement. To accurately assess the impact of the virtual laboratory, the study controls for factors such as student age, prior knowledge, family background, computer skills, and teacher characteristics. This ensures that the effect of the virtual laboratory on academic performance, which is measured through grades, test scores, and overall comprehension, is isolated and precisely evaluated.

## Methods

### Study Area

Dilla University, a public institution located in Dilla town in Ethiopia’s southern region, is often called the “University of the Green Land” due to the abundant greenery in the surrounding Gedeo area. The University traces its roots back to the establishment of the Dilla College of Teachers’ Education and Health Science in 1996. Over time, the Dilla campus expanded to include 12 departments and achieved independent university status in 2006 (decree number 129/1999). Currently, technology-based instructional methods are used at the university to facilitate and enhance the teaching and learning processes.

### Research Design and Paradigm

Under the postpositivist paradigm, this study used a quasi-experimental research design to investigate the effect of a virtual chemistry laboratory on the academic achievements of fourth-year undergraduate chemistry students in their first semester at Dilla University. Quasi-experimental research designs allow researchers to explore cause-and-effect relationships and compare groups under different circumstances or treatments, aiming to establish causality between an intervention and an outcome, as well as identify links between independent and dependent variables. The study was conducted through the steps listed in the following subsections.

#### Step 1: Group Assignment

Fourth-year undergraduate chemistry students in their first semester at Dilla University were divided into treatment (virtual laboratory) and control (real laboratory and lecture) groups based on factors such as academic performance, family background, technology skills, age, and gender.

#### Step 2: Test Preparation and Pretest Measurement

A total of 6 experiments, comprising 12 subexperiments, were selected from the practical organic chemistry course and designed for a simulated laboratory setting. A test was prepared based on these experiments. Before any treatment or intervention, the real, virtual, and lecture-only groups were assessed with a pretest to establish their baseline knowledge.

#### Step 3: Intervention

The experiments were taught using three different methods: (1) the real laboratory group learned the theory and conducted experiments in a real laboratory, (2) the lecture-only group learned both theory and experiments solely through lectures, and (3) the virtual laboratory group learned the theoretical aspects and performed experiments in a virtual laboratory setting.

#### Step 4: Posttest Measurement

After completing the selected experiments in a chemistry practical course, all groups took a posttest (ie, the semester final) to assess changes in the dependent variable.

#### Step 5: Comparison of Results

Comparisons were made among the real, virtual, and lecture-only groups based on their practical chemistry scores, both before and after the intervention. The hypotheses in this study were examined based on the tests administered following the outlined steps, and the analysis of the results was conducted using the prescribed analytic tools.

### Target Population and Samples

The target population for this study consisted of undergraduate students at Dilla University, specifically those enrolled in the chemistry department of the College of Computational and Natural Sciences. This group was chosen because they were enrolled in practical chemistry courses during the research, making them ideal candidates to evaluate the effectiveness of a virtual laboratory.

For this study, fourth-year students at Dilla University, who were enrolled in the BSc chemistry program, were selected as the sample. The program had 2 sections: section 1 consisted of 20 students and section 2 consisted of 40 students, leading to a total sample size of 60 students. To assess the impact of the intervention on academic performance, these students were divided into control (real laboratory and lecture only) and experimental (virtual laboratory) groups. The real laboratory group, consisting of 20 students, conducted experiments using the real laboratory method; the lecture-only group, consisting of 20 students, followed a lecture-only method; and the virtual laboratory group, consisting of 20 students, used the virtual laboratory method. The outcomes of this study’s hypothesis depended on the performance of these 60 students. The selected participants from each section of the university are summarized and presented in Table 1.

**Table 1:** Study sample distribution, Dilla University, 2024.

Group	BSc in chemistry: section 1 (n=20), n (%)	BSc in chemistry: section 2 (n=40), n (%)	Total (n=60), n (%)
Real laboratory	7 (35)	13 (32)	20 (33)
Lecture only	6 (30)	14 (35)	20 (33)
Virtual laboratory	7(35)	13 (33)	20 (33)

### Data Source and Data Gathering Instruments

The research used primary data sources, ensuring that the information collected was recent and directly aligned with the study objectives. Using primary data is essential in educational research as it provides context-specific data, leading to more accurate and insightful analysis [26].

In this study, both objective and subjective examination items were used to gather relevant data from the students. The objective section included 26 multiple-choice questions and 9 fill-in-the-blank questions, which provided quantifiable data to assess students’ academic performance [27]. In addition, the subjective section consisted of 5 short-answer questions, enabling a detailed evaluation of students’ cognitive abilities and practical knowledge [28]. Overall, the results of the research hypotheses were based on the data collected from the administered tests.

### Validity and Reliability of the Study Instrument

To ensure that the measure is the intended construct (construct validity) and to confirm that the test covers relevant content (content validity), the tests were evaluated by subject teachers and experts. Following feedback from subject teachers and experts, several items in the test were rearranged, revised, removed, and reassessed. A pilot test was conducted with 20 students outside the research area to assess the reliability and consistency of the test results. Following the initial test, the same group underwent the test again 2 weeks later to ensure consistency. The study used Pearson correlation coefficient analysis on the collected data, revealing a Pearson correlation coefficient of 0.88. According to Cohen [29] a correlation in the range of 0.50 to 1.00 indicates a strong relationship, signifying that the test results remained highly reliable and consistent over the 2-week period. This strong correlation confirms the stability of the test scores and highlights the overall reliability of the test, which supports the validity of the research hypothesis results.

### Data Collection Procedure

An official letter explaining the intent of the study was obtained from the chemistry department at Hawassa University, and it was provided to the head of the chemistry department at Dilla University. The purpose and the objective of the study were clearly explained and communicated to the department head, staff, laboratory technicians, and students before beginning the study. A total of 6 experiments (comprising 12 subexperiments) that can be performed in a real laboratory were selected from Practical Organic Chemistry III courses (course code Chem-444) with the course instructor and the laboratory assistant in Dilla University. The subject teachers evaluated the experiments and provided the necessary corrections before the commencement of the study. The selected experiments were designed by a reaction simulation software for the virtual laboratories. The software assessment was performed for fourth-year students at Kotebe University, Addis Ababa, before the software was implemented to research participants in Dilla University. The software was modified by the developer to accommodate the feedback given by the students and the course instructors. The research participants received training on how to implement the virtual chemistry laboratory software. The test was prepared based on the 6 experiments (comprising 12 components). At the beginning, a pretest was administered to assess the students’ baseline knowledge. The students learned the experiments in 3 different methods, namely, real laboratory, virtual laboratory, and lecture only. The experiment was conducted on a weekly basis for a duration of 6 weeks. When they finished learning the designed experiments in these methods, a posttest was administered to evaluate the students’ understanding. The data collected in this study consisted of test scores, which were analyzed using SPSS (version 26; IBM Corp).

### Experiments Performed by Participants

#### Experiment 1: Chemical Tests for Alkenes and Alkynes

##### Bromine Solution Test

Alkenes and alkynes were tested by adding bromine solution to the sample. The disappearance of the reddish-brown color of bromine indicated the presence of a carbon-carbon double bond (alkene) or triple bond (alkyne) due to the addition reaction between bromine and the unsaturated carbon atoms.

##### Potassium Permanganate (Baeyer) Test

A potassium permanganate solution was used to test for the presence of unsaturation (alkenes and alkynes). A positive result was indicated by the decolorization of the purple permanganate solution and the formation of a brown precipitate of manganese dioxide, signifying an oxidation reaction at the double or triple bond.

#### Experiment 2: Chemical Tests for Aromatic Hydrocarbons Without Functional Groups

##### Sulfuric Acid Test

Aromatic hydrocarbons were subjected to concentrated sulfuric acid. Aromatic rings, being highly stable, undergo electrophilic substitution rather than addition. The formation of sulfonated products (such as benzene sulfonic acid) demonstrated the presence of an aromatic hydrocarbon.

##### Chloroform and Aluminum Chloride Test

Aromatic hydrocarbons were tested by adding chloroform and aluminum chloride. The Lewis acid aluminum chloride acted as a catalyst in Friedel-Crafts alkylation or acylation reactions. A color change indicated interaction with the aromatic system, confirming its presence.

#### Experiment 3: Identification of Alcohol Functional Groups

##### Jones Oxidation Test

Alcohols were tested using the Jones reagent (chromic acid in dilute sulfuric acid). Primary alcohols were oxidized to carboxylic acids, and secondary alcohols were oxidized to ketones, as evidenced by a color change from orange to green due to the reduction of chromium (VI) to chromium (III). Tertiary alcohols did not react, as they are resistant to oxidation under these conditions.

##### Lucas Test

The Lucas test was used to distinguish between primary, secondary, and tertiary alcohols. A mixture of the alcohol and Lucas reagent (concentrated hydrochloric acid and zinc chloride) was added. Tertiary alcohols reacted quickly, forming an insoluble alkyl chloride, whereas secondary alcohols reacted more slowly, and primary alcohols showed little to no reaction.

##### Liebermann Nitroso Reaction for Phenol

This test was used to detect phenolic groups in alcohols. Phenols reacted with sodium nitrite and concentrated sulfuric acid to form a deep blue or green color, confirming the presence of phenolic groups in the compound.

#### Experiment 4: Chemical Tests for Aldehydes and Ketones

##### 2,4-Dinitrophenylhydrazine Test

Aldehydes and ketones were identified by adding the 2,4-DNPH reagent. The formation of a yellow, orange, or red precipitate (2,4-dinitrophenylhydrazone) confirmed the presence of a carbonyl group (C=O) in aldehydes or ketones.

##### Tollen Test (Silver Mirror Test)

Aldehydes were further differentiated from ketones using the Tollen reagent (ammoniacal silver nitrate). A positive test was marked by the formation of a silver mirror on the walls of the test tube, indicating the oxidation of the aldehyde to a carboxylic acid and the reduction of silver ions to metallic silver. Ketones did not give a positive result in this test.

#### Experiment 5: Chemical Test for Amines

For the nitrous acid test, amines were tested with nitrous acid (generated in situ from sodium nitrite and hydrochloric acid). Primary aliphatic amines reacted to form nitrogen gas and an alcohol, while primary aromatic amines formed diazonium salts. Secondary amines formed nitrosamines, and tertiary amines showed no reaction, allowing differentiation between amine types based on reaction products.

#### Experiment 6: Chemical Tests for Carboxylic Acids

##### Sodium Bicarbonate Test

The presence of carboxylic acids was confirmed by adding sodium bicarbonate (NaHCO₃). Carboxylic acids reacted with the base to produce carbon dioxide gas, which was observed as effervescence, indicating the acidic nature of the compound.

##### Silver Nitrate Solution (Ethanolic) Test

Carboxylic acids were further tested by reacting with silver nitrate in ethanol. The formation of a precipitate indicated the presence of a carboxyl group, as carboxylic acids formed silver salts with the silver ions in the solution.

### Implementation of Lesson Intervention

In the study, all 3 groups took a pretest before the intervention began. The intervention was conducted using 3 different teaching methods. The real laboratory group learned theoretical concepts and performed experiments in a real laboratory. The lecture group learned both theory and experiments solely through lectures. The virtual laboratory group learned the theoretical part and conducted experiments using a virtual laboratory. The intervention lasted 1 semester, from October 2023 to January 2024, with weekly experimental sessions. The time required to complete each experiment varied by teaching method: an average of 3 hours in the real laboratory, 2 hours in the virtual laboratory, and 1.5 hours at the lectures.

### Data Analysis Tools and Techniques

Data analysis was performed using SPSS, incorporating both descriptive and inferential statistical methods. Descriptive statistics, including percentages, means, and SDs, were used to summarize the data and provide an overview of the results. To explore the differences between groups, inferential statistics were used. One-way ANOVA was used to assess variations in pre- and posttest results across the 3 independent groups. The Tukey honestly significant difference (HSD) test was applied for post hoc analysis to compare mean scores among the groups following the ANOVA. Furthermore, an independent-sample *t* test (2-tailed) was conducted to examine differences in posttest results between male and female students within the virtual laboratory group, providing insights into gender-based performance variations.

### Ethical Considerations

The study protocol was reviewed and approved by the Hawassa University Ethics Review Committee (CNCS-REC030/23). In addition, the chemistry department at Dilla University provided a letter of support for the research. All participants received detailed information regarding the purpose, nature, and potential implications of the study. After being fully informed, participants provided verbal consent to participate in the study. This approach was approved by the ethics committee, and documentation of verbal consent was recorded as per the committee’s guidelines. Participants were informed of their right to withdraw from the study at any time without any consequences or impact on their academic standing. Confidentiality and anonymity were strictly maintained throughout the research process. All data collected were deidentified, and no personal information that could reveal the participants’ identities was recorded. Data were securely stored in password-protected files and were accessible only to the research team. Participants’ identities were protected in all reports and publications stemming from the research. No financial compensation or incentives were provided to participants for their involvement in the study. Participation was voluntary, and participants were informed that their decision to participate or withdraw would not affect their academic evaluations. The assessments and data collected during the study were used solely for research purposes and did not influence the universities’ grading or result systems. The study was designed to assess the impact of virtual laboratories on students’ achievement without affecting their academic grades or outcomes.

## Results

### Respondents’ Profile Analysis

As depicted in Table 2, most participants (52/60, 87%) were males, aged between 21 and 23 years, with cumulative grade point averages (CGPAs) ranging from 2.6 to 3.5. Most participants had medium computer proficiency and came from families with nearly equal proportions of educated and uneducated members. Overall, the profile of the participants implied that the study’s findings reflect the experiences of a diverse group of students in terms of gender, age, academic performance, computer proficiency, and family educational background. This diversity can enhance the validity and applicability of the research outcomes, offering valuable insights for educational institutions aiming to implement virtual laboratories in similar contexts.

**Table 2:** Respondents’ profile, Dilla University, 2024.

Variables		Male participants (n=52), n (%)	Female participants (n=8), n (%)
Age (y)			
	18-20	0 (0)	0 (0)
	21-23	38 (63)	8 (13)
	24-27	13 (22)	0 (0)
	≥28	1 (2)	0 (0)
CGPA^a^			
	2.00-2.50	8 (13)	4 (7)
	2.51-3.00	23 (38)	2 (3)
	3.01-3.50	18 (30)	2 (3)
	3.51-4.00	3 (5)	0 (0)
Computer skill			
	High	1 (2)	0 (0)
	Medium	49 (82)	7 (12)
	Low	2 (3)	1 (2)
Family background			
	Educated family	26 (43)	4 (7)
	Uneducated family	26 (43)	4 (7)

^a^CGPA: cumulative grade point average.

### Hypothesis 1

#### Analysis of Pretest Results

As depicted in Table 3, the mean scores for the real laboratory group (mean 31.7, SD 7.2), lecture group (mean 31.6, SD 7.6), and virtual laboratory group (mean 31.4, SD 10.1) are quite similar, indicating no statistically significant differences among the groups. However, the SDs for the groups suggest moderate variability within each group. The SEs, reflecting the precision of the sample means, are smallest for the real laboratory group (SE 1.60) and largest for the lecture group (SE 2.26), indicating greater variability in the lecture group’s scores. The 95% CIs, ranging from 28.4 to 35.1 for the real laboratory group, 26.9 to 36.4 for the lecture group, and 27.8 to 34.9 for the virtual laboratory group, show a significant overlap, supporting the conclusion that there are no statistically significant differences between the groups. The minimum scores for the real laboratory group, lecture group, and virtual laboratory group are 17, 15, and 17, respectively, and the maximum scores for the real laboratory group, lecture group, and virtual laboratory group are 50, 52, and 50, respectively. These scores are relatively consistent across the groups, reinforcing that initial academic performance was comparable between the groups. To further confirm whether the mean pretest scores across the 3 groups were comparable, an ANOVA was conducted.

**Table 3:** Variability in pretest scores of the 3 independent categories, Dilla University, 2024.

Group	Scores, mean (SD)	Scores, range	Scores, SE	Scores, 95% CI
Real laboratory (n=20)	31.7 (7.2)	17-50	1.60	28.4-35.1
Lecture only (n=20)	31.6 (10.1)	15-52	2.26	26.9-36.4
Virtual laboratory (n=20)	31.4 (7.6)	17-50	1.71	27.8-34.9

The ANOVA results in Table 4 reveal that the between groups sum of squares is 1.458, with 2 df, yielding a mean square between groups of 0.729. This low value suggests that the group means are relatively similar to one another. In contrast, the sum of squares within groups is much larger at 4035.625, with 57 df, resulting in a mean square within groups of 70.800. This indicates that most of the variation in pretest scores occurs within the groups rather than between them. The computed *F* value (*F*_2,57_=0.010) is far below the critical value of 4.001, showing no significant differences in mean scores among the groups. In addition, the *P* value of .99 is well above the standard significance level of .05, further confirming that there is no statistically significant variations in the group mean scores.

**Table 4:** One-way ANOVA results comparing pretest scores of the 3 independent groups, Dilla University, 2024.

	Sum square	Mean square	*F* test (*df*)	*P* value
Between group	1.458	0.729	0.010 (2)	.99
Within group	4035.625	70.800	0.010 (57)	.99
Total	4037.083	—^a^	0.010 (59)	.99

^a^Not applicable.

#### Analysis of Posttest Result

As depicted in Table 5, the real laboratory group achieved the highest mean score (mean 62.6, SD 10.7), indicating that students who engaged in hands-on laboratory activities performed better overall. The relatively low SD suggests that most students in this group had scores close to the mean, reflecting consistent performance. The SEM (SEM 2.4) further supports the reliability of this group’s average score. In contrast, the lecture-only group had the lowest mean score (mean 43.7, SD 11.5), indicating that relying solely on lectures was the least effective method for enhancing students’ understanding and application of the material. The SD shows a broader range of scores, reflecting greater variability in students’ performance. The higher SEM (SEM 2.6) suggests less precision in the average score, indicating more dispersed student scores. The virtual laboratory group, with a mean score of 55.5 (SD 6.8), falls between the real laboratory and lecture-only groups. The relatively low SD and the smallest SEM (SEM 1.5) among the 3 groups indicate consistent performance and precision in the average score. Overall, these results demonstrate differences in mean scores between the groups. To further confirm these mean differences, a 1-way ANOVA was conducted.

**Table 5:** Descriptive statistics results of the posttest scores for the 3 independent groups, Dilla University, 2024.

Learning style	Scores, mean (SD; range)	Scores, SEM	Scores, sum
Real laboratory (n=20)	62.6 (10.7; 45-80)	2.4	1252.5
Lecture only (n=20)	43.7 (11.5; 20-65)	2.6	875.0
Virtual laboratory (n=20)	55.5 (6.8; 40-65)	1.5	1110.0
Total (n=60)	53.9 (12.5; 20-80)	1.6	3237.5

The ANOVA results presented in Table 6 show that the sum of squares between groups is 3633.958, with 2 df, resulting in a mean square between groups of 1816.979. This high value suggests substantial variation in posttest scores among the real laboratory, virtual laboratory, and lecture-only groups. Conversely, the sum of squares within groups is 5619.688, with 57 df, leading to a mean square within groups of 98.591, indicating considerable variation within each group. The calculated *F* value (*F*_2,57_=18.429) exceeds the critical value of 4.001, indicating significant differences in mean scores among the groups. In addition, the *P* value of <.001 is well below the standard significance level of .05, confirming that the differences in group mean scores are statistically significant. Overall, these results suggest that there is a difference in mean scores among the 3 independent groups in the posttest. To determine which group’s mean differs from the others and by how much, the Tukey HSD post hoc test was used for pairwise comparisons.

**Table 6:** One-way ANOVA results comparing the posttest scores of the 3 independent groups, Dilla University, 2024.

	Sum square	Mean square	*F* test (*df*)	*P* value
Between group	3633.958	1816.979	18.429 (2)	<.001
Within group	5619.688	98.591	18.429 (57)	<.001
Total	9253.646	—^a^	18.429 (59)	<.001

^a^Not applicable.

In Table 7, the Tukey HSD post hoc test results reveal that the mean difference between the real laboratory and lecture-only groups is 18.88, indicating that the real laboratory group scored, on average, 18.88 points higher than the lecture-only group. The significance value (*P*<.001) is less than the .05 threshold, confirming that this difference is statistically significant. In addition, the 95% CI value (11.3190-26.4310) does not include 0, further supporting the significance of this finding. Overall, the real laboratory method is significantly more effective than the lecture-only method. In comparison, the mean difference between the real laboratory and virtual laboratory groups is 7.12, suggesting that the real laboratory group scored 7.12 points higher, on average, than the virtual laboratory group. However, the *P* value of .07 is slightly above the .05 significance threshold, indicating that this difference is not statistically significant. The 95% CI value (–0.4310 to 14.6810) crosses 0, further suggesting no significant difference between the real laboratory and virtual laboratory methods. Therefore, there is no statistically significant difference in effectiveness between the real laboratory and virtual laboratory methods. Finally, the mean difference between the virtual laboratory and lecture-only groups is 11.7, indicating that the virtual laboratory group scored, on average, 11.7 points higher than the lecture-only group. The *P* value of .001 is below the .05 significance level, confirming that this difference is statistically significant. The 95% CI value (4.1940-19.3060) also does not include 0, further confirming the significance. Thus, the virtual laboratory method is significantly more effective than the lecture-only method.

**Table 7:** The Tukey honestly significant difference post hoc test comparing specific group means, Dilla University, 2024.

Laboratory method		Scores, mean difference (SE)	*P* value	Range
**Real laboratory**				
	Lecture only	18.87 (3.13)	<.001	11.3190 to 26.4310
	Virtual laboratory	7.12 (3.13)	.07	–0.4310 to 14.6810
**Lecture only**				
	Real laboratory	–18.87 (3.13)	<.001	–26.4310 to –11.3190
	Virtual laboratory	–11.75 (3.13)	.001	–19.3060 to –4.1940
**Virtual laboratory**				
	Real laboratory	–7.12 (3.13)	.07	–14.6810 to 4310
	Lecture only	11.75 (3.13)	.001	4.1940 to 19.3060

### Hypothesis 2: Analysis of Posttest Scores of the Virtual Laboratory Group

**Figure 2:**
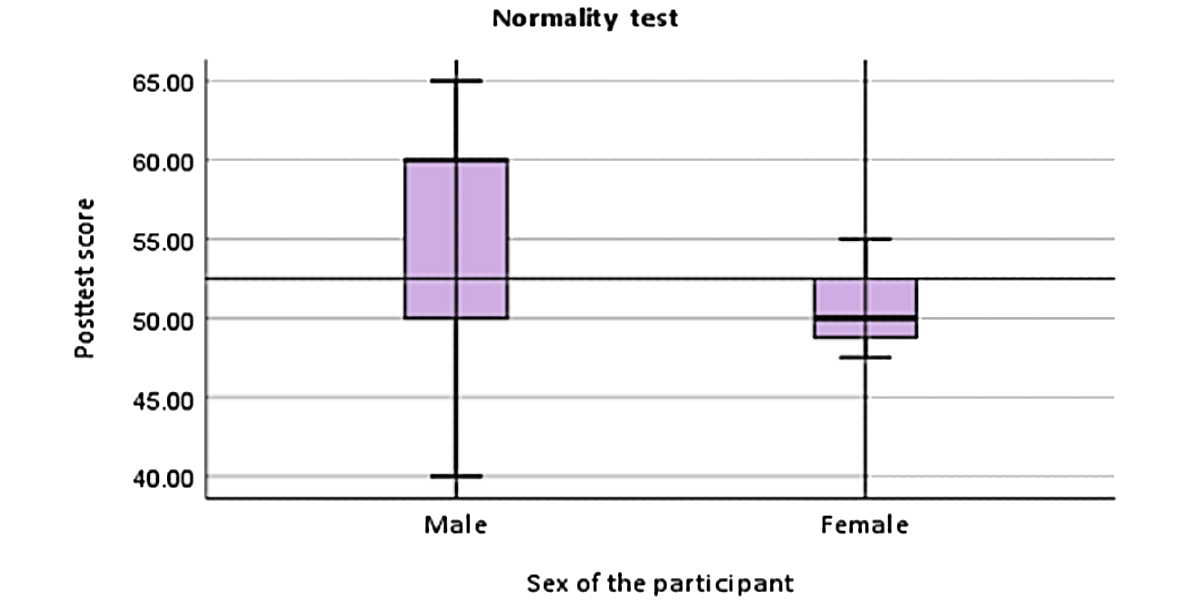
Normality test of posttest scores by gender in the virtual laboratory group.

Although an independent-sample *t* test can be conducted with this sample, the small size may affect the reliability of the results. Nevertheless, verifying normality is essential to ensure the test’s accuracy. As shown in Figure 2, the median posttest score for male students is approximately 55, while for female students, it is slightly >50, suggesting that male students performed better on average. The IQR for male students (50-60) shows greater variability compared with female students (48-52). Male scores range from 40 to 65, indicating a broader spread, while female scores are more concentrated between 45 and 54. Overlapping score ranges suggest no significant performance difference between genders. Overall, the box plots for both groups show a relatively symmetrical distribution, with the medians centered within the IQRs. The lack of outliers and balanced whiskers further support the assumption of normality. Therefore, the data meet the assumptions for conducting an independent-sample *t* test, assuming that the homogeneity of variances is also satisfied.

As shown in Table 8, the Levene test for equality of variances yielded *F*_18,4.8_=1.977 and *P*=.18. As the *P* value is >.05, this suggests that the variances between the male and female posttest scores are approximately equal. The *t* test value, assuming equal variances, is 1.310, with a 2-tailed *P* value of .21. Because the *P* value exceeds .05, there is no statistically significant difference between the posttest scores of male and female students in the virtual laboratory group. The mean difference between the male and female students’ posttest scores is 5.49, with male students scoring higher on average. However, this difference was not statistically significant. The 95% CI for the mean difference ranges from –3.31 to 14.29, and because this interval includes 0, it further suggests that the difference in scores between the 2 groups could be due to chance. Overall, this result suggests that gender did not have a substantial impact on students’ performance in the virtual laboratory setting.

**Table 8:** Independent-sample t test comparing the posttest scores of male and female students.

	Levene test for equality of variances		*t* test for equality of means			
	*F* test (*df*)	*P* value	*t* test (*df*)	*P* value (2-tailed)	Mean difference	SE difference (95% CI)
Equal variances assumed	1.97 (1,18)	.20	1.31 (18)	.21	5.49	4.19 (–3.311 to 14.292)
Equal variances not assumed	—^a^	—	1.97 (4.8)	.11	5.49	2.77 (–1.726 to 12.706)

^a^Not applicable.

## Discussion

### Overview

This study investigated the impact of virtual laboratories on undergraduate students’ academic achievement in a practical chemistry course. The results showed that while all groups (real laboratory, virtual laboratory, and lecture only) had comparable performance levels before the intervention, the posttest scores revealed significant differences. The real laboratory group demonstrated the highest achievement, followed by the virtual laboratory group, while the lecture-only group performed the lowest. The findings suggest that hands-on laboratory experiences are the most effective methods for enhancing student learning, although virtual laboratories provide a valuable alternative, particularly when physical resources are limited. In addition, no significant gender differences were found in the virtual laboratory group’s performance, indicating that both male and female students benefited equally from virtual laboratory experiences.

### Principal Findings

#### Pretest Performance

The pretest scores for the real laboratory, virtual laboratory, and lecture-only groups were very similar (mean 31.7, 31.6, and 31.4, respectively), with no statistically significant differences (*F*_2,57_=0.010; *P*=.99). This indicates that all groups started at comparable performance levels before the instructional interventions, supporting the premise that any subsequent differences in achievement are not due to preexisting differences in student ability. Overlapping 95% CIs further indicated no significant differences in baseline performance among the 3 groups.

#### Posttest Performance

The real laboratory group attained the highest mean posttest score of 62.6, followed by the virtual laboratory group with a mean score of 55.5 and the lecture-only group with a mean score of 43.7. The ANOVA analysis showed a significant variation in performance among the groups (*F*_2,57_=18.429; *P*<.001). Hands-on laboratory work led to the best learning outcomes, but virtual laboratories were also more effective than the lecture-only approach. This highlights the importance of direct interaction with physical materials and equipment in enhancing students’ understanding and retention of scientific concepts. The finding also underscores that virtual laboratories are a valuable supplement when real laboratory resources are limited, effectively bridging hands-on experience gaps and simplifying abstract scientific concepts for students.

#### Post Hoc Analysis (Tukey HSD)

The real laboratory method significantly outperformed the lecture-based method (mean difference 18.88; *P*<.001). This emphasizes the superior effectiveness of hands-on laboratory activities compared to lectures for enhancing student learning. The difference between the real laboratory and virtual laboratory methods was not statistically significant (mean difference 7.12; *P*=.07), indicating similar educational benefits. Furthermore, students in the virtual laboratory group achieved significantly higher scores than those in the lecture-only group (mean difference 11.75; *P*=.001). This indicates that virtual laboratories can significantly enhance learning outcomes compared with lecture-only methods. They offer a valuable alternative by providing an interactive and experiential learning experience that deepens the understanding and retention of scientific concepts.

#### Gender Comparison in the Virtual Laboratory Group

The independent-sample *t* test revealed no significant difference in posttest scores between male and female students in the virtual laboratory group (t_18_=1.310; *P*=.21), with a mean difference of 5.49 points. This indicates that gender does not influence performance in virtual laboratory settings. Overall, the results suggest that both male and female students benefit equally from virtual laboratory experiences.

### Comparison With Prior Work

Gungor et al [19] observed that prior knowledge did not significantly influence student performance when various teaching methods were used, a finding that aligns with the comparable pretest scores observed in this study. This consistency suggests that the effectiveness of teaching methods, rather than students’ prior knowledge, plays a more significant role in shaping educational outcomes.

Shana and Abulibdeh [8] and Ekwueme et al [11] highlighted the crucial role of hands-on learning, aligning with the superior performance observed in the real laboratory group. Their findings indicated that direct interaction with materials significantly enhances the understanding of scientific concepts. Similarly, Abate Jote [6] noted that students in traditional laboratory settings excelled in standardized processes and memorization-based calculations due to the repetitive and structured hands-on experiments. This consistent application of established methods improved their procedural knowledge and rote memorization, further supporting the superior performance of the real laboratory group.

Tatli and Ayas [9] reported that virtual laboratory software can be as effective as traditional laboratories in improving student learning, aligning with this study’s finding that there was no statistically significant difference between the real laboratory and virtual laboratory groups. In addition, Darrah et al [30] found that virtual laboratories in university settings can be as effective as traditional hands-on laboratories. This aligns with the findings of this study, which showed no statistically significant difference in performance between the real laboratory and virtual laboratory groups, indicating that both methods offer comparable educational benefits.

The absence of significant gender differences in performance is reinforced by studies such as those by Famuwagun and Ojobola [4] and Akomaye [18], which highlighted that virtual laboratories create an equitable learning environment, enabling both male and female students to perform equally well. Similarly, studies by Pal [31] and Oladejo et al [32] found that both male and female students achieved comparable levels of success when virtual laboratory–based active learning methods were used for instruction. This aligns with this study’s findings, where male and female students achieved similar scores in the virtual laboratory.

### Limitations

Although the study offers valuable insights into the effectiveness of virtual laboratories, several limitations should be acknowledged. First, the sample size was limited to 60 students, which may restrict the generalizability of the findings to broader populations. In addition, the study focused solely on Dilla University in southern Ethiopia, which may limit the applicability of the findings to other institutions or regions where conditions, resources, and student demographics may vary significantly, such as in terms of socioeconomic backgrounds, access to technology, and prior educational experiences. Furthermore, the study centered on a specific chemistry course, which may not adequately reflect the effectiveness of virtual laboratories in other courses or disciplines, particularly those requiring different levels of abstraction or types of practical skills.

### Conclusions

This study highlights the effect of virtual laboratories on undergraduate chemistry education. Initially, students in real laboratory, virtual laboratory, and lecture-only groups had similar baseline knowledge, indicating that teaching methods, rather than initial proficiency, shape learning outcomes. Posttest results revealed that the real laboratory group scored higher than both the virtual laboratory and lecture-only groups, underscoring the enhanced understanding and retention achieved through hands-on real laboratory experiences. However, virtual laboratories proved to be a valuable supplementary tool, offering significant advantages over lecture-only methods and providing benefits comparable to real laboratories when physical laboratory access is limited. Gender did not significantly influence performance in virtual laboratories, emphasizing their equitable learning environment. Overall, while real laboratories are crucial for deepening scientific understanding, computer-based laboratories offer flexibility and accessibility, making them an effective complement in resource-constrained settings. Combining both virtual and real laboratories can enhance the educational experience in practical chemistry and other experimental sciences.

## Abbreviations

CGPA: cumulative grade point average
HSD: honestly significant difference

## Data Availability

The datasets generated and analyzed during this study are not publicly available due to privacy or confidentiality concerns but are available from the corresponding author on reasonable request.
